# A New Approach to Diagnosis to Posterior Cross Bite: Intraoral Photography and Wala Ridge

**DOI:** 10.3390/ijerph19159443

**Published:** 2022-08-01

**Authors:** Rocío Ramón, Alberto Adanero, Mónica Miegimolle

**Affiliations:** Clinical Dentistry Department, Biomedical and Sciences Faculty, European University of Madrid, Villaviciosa de Odón, 28670 Madrid, Spain; rocioramonherrero@hotmail.com (R.R.); monica.miegimolle@universidadeuropea.es (M.M.)

**Keywords:** posterior crossbite, Wala Ridge, transverse pathology

## Abstract

A posterior crossbite is an occlusion disorder that occurs in the transverse plane. It occurs when the buccal cusps of the upper premolars and molars engage lingually with the buccal cusps of the lower teeth. It can be unilateral or bilateral (involving one or more teeth) in the primary, mixed, or permanent dentition. A crossbite may appear in early dentition stages and it can be dental or functional. It can lead to skeletal crossbite in mixed dentition. Therefore, early diagnosis and treatment are crucial. Material and methods: The selected sample included 204 patients in growing stage divided into two groups: a study group of 102 patients with posterior crossbite and a control group of 102 patients without malocclusion. To analyze the pathology, intraoral frontal photographs and study models were taken, in which the bone component was measured from the Wala Ridge. Results: The use of the photographs to study the Wala Ridge was confirmed. The mean maxillary width was 57.8 mm (SD 1.7) and mandibular width was 56.4 mm (SD 1.7) for the control group, with a maxillomandibular difference of 1.4 mm (SD 0.7); and 52.7 mm (SD 3.7) and 55.5 mm (SD 3.6), respectively, with a maxillomandibular difference of −2.8 mm (SD 1.4) for the study group. A higher maxillomandibular discrepancy was observed in patients with a posterior crossbite that involved more than one tooth in addition to the permanent first molar. It was also higher in patients with bilateral posterior crossbite. Conclusions: Intraoral frontal photography can be used as a diagnostic method to measure the maxillomandibular difference using the Wala Ridge.

## 1. Introduction

Malocclusions have constituted one of the main public health problems, being the third most prevalent problem in the world population after dental caries and periodontal disease according to the WHO [1].

Malocclusions occur in the three planes of space, and a simplified method of classifying them is provided by different authors as follows [2,3,4,5]:

- Sagittal plane: Class I, Class II and Class III with their divisions and sub-divisions;
- Vertical plane: Open bite or deep bite;
- Transverse plane: Crossbite or scissor bite.

The first classification of malocclusions by Angle in 1899 was an influential factor in the development of orthodontics, as he identified malocclusion as the deviation of normal growth and development of dentition [6]. Please note that this classification considers only sagittal directions, which explains why it has been considered incomplete as it fails to classify malocclusions transversally and vertically [7].

Ackerman and Proffit [8] completed Angle’s classification in 1960, covering the anteroposterior, vertical, and transverse planes.

During the last two decades, an important sector of the orthodontic community has shown great interest in the early treatment of malocclusions, understood as the correction of skeletal, dentoalveolar, and muscular discrepancies, whether existing or in the process of development, with the intention of preparing a better orofacial environment before the eruption of the permanent dentition has been completed. By starting therapeutic treatments at an earlier age, the need for complex orthodontic treatment is minimized or eliminated [9,10].

A posterior crossbite is an occlusion disorder that occurs in the transverse plane. Brodie described it in 1943 as “the anomaly wherein one or more teeth of the posterior sector meet in a vestibulolingual fashion with one or more teeth of the opposing arch” [11]. Treatment of this crossbite has been studied for many years [12,13], and we are going to focus on the diagnosis.

To achieve harmonious growth, Will Andrews and his son worked to develop the diagnostic philosophy of “the 6 elements of orofacial harmony.” “Element III,” aims to analyze the transverse ratio of the maxilla and mandible, based on bony and dental baselines [14,15,16].

L.F. Andrews and W.A. Andrews [17] suggested the use of an anatomical baseline as a parameter to centralize the roots of the teeth in the basal bone, which they called the Wala Ridge.

The Wala Ridge is the band of soft tissue immediately superior to the mucogingival junction of the mandible, at or near the line passing through the centers of rotation of the teeth; it is exclusive to this bone. The mandibular dental arch will have the ideal shape when the midpoint of the vertical axes of the facial surfaces (“AF” points) of the central and lateral incisors, canines, first premolars, second premolars, first and second molars are 0.1 mm, 0.3 mm, 0.6 mm, 0.8 mm, 1.3 mm, 2.0 mm and 2.2 mm, respectively, from the Wala Ridge.

In the transverse dimension, when the jaws do not relate optimally, usually due to a deficiency in jaw width, the teeth will erupt in a crossbite or reconfigure their inclinations to avoid a crossbite. This compensation involves a lingual inclination of the posterior mandibular teeth, which are then described as excessively negatively inclined. In addition, the maxillary posterior teeth are easily inclined, described as excessively positively inclined [18].

It is important to make a correct diagnosis by analyzing whether the patient has pathology in the sagittal, vertical, or transverse plane. In child patients, it is essential to focus on any skeletal problems because, as they grow, an early diagnosis can eliminate the problem and direct growth to the patient’s needs.

In the case of a transverse problem, after completing the adolescent growth spurt, the palatal suture ossifies over time, both bones fuse gradually, so the forces to be applied will be more intense to produce a significant maxillary skeletal expansion. In addition, there are different studies that show that there is no spontaneous correction of the posterior crossbite during growth. [19]. Therefore, it is vitally important to assess the craniofacial skeleton in the transverse dimension as early as possible and accurately diagnose the need for early maxillary transverse expansion to improve the efficiency and effectiveness of treatment [20].

Nowadays, the objective of a good diagnosis is based on being able to use the maximum of available tools within our reach. The intraoral frontal photograph gives us great information at the bone level. In addition to study models and 2D and 3D radiographs, we can use photography to obtain information more easily, avoiding radiation. We are then able to create a detailed plan for treatment.

- The use of frontal intraoral photography allowed a perfect initial evaluation and a better understanding of the patient’s treatment plan;
- The intraoral frontal photography technique is a predictable and minimally invasive technique that helps to reduce labor time and potential errors commonly associated with measurements on 2D radiographs or study models [21].

### 1.1. Aims

#### 1.1.1. Primary Aim

- To analyze the possibility of using intraoral frontal photography as a diagnostic method to measure the maxillary–mandibular difference from the Wala Ridge, comparing it with the measurements taken on the study models.

#### 1.1.2. Secondary Aims

- Analyze the maxillary width of the entire sample from the Wala Ridge;
- Analyze the mandibular width of the entire sample from the Wala Ridge;
- Know the mean maxillomandibular difference in the sample analyzed;
- Estimate whether there are differences in maxillomandibular size between genders in the study group;
- Observe if, in the study group, there are differences in maxillomandibular size according to their dentition stage;
- Assess whether the maxillomandibular difference in the study group is greater if it affects only the first permanent molar or if it affects more than one tooth;
- Assess whether the maxillomandibular difference is greater in patients with unilateral or bilateral, right or left posterior crossbite.

## 2. Materials and Methods

A single researcher has carried out the study with records belonging to patients aged between 6 and 12 years of age. A total of 204 patients divided into two groups were used: a study group, belonging to patients with posterior crossbite and a control group, without posterior crossbite. Parents or guardians were given a document where they could authorize the use of the records in accordance with the data protection law, together with the informed consent of the project. The study was conducted in accordance with the Declaration of Helsinki, and approved by the Institutional Ethics Committee of European University of Madrid (protocol code CIPI/19/168, approved on April 2019).

The study included patients who attended the Dental University Clinic of the Advanced Orthodontics Master program at Universidad Europea de Madrid, as well as pediatric dentistry and orthodontic patients from the International Dental Institute of Alicante.

### 2.1. Study Group Population

A—Inclusion Criteria

- Child patients of either sex aged 6 to 12 years;
- Patients with complete medical records;
- Children with mixed primary and permanent dentition stage;
- Emergence of the first four permanent molars;
- Presence of posterior crossbite in patients.

B—Exclusion Criteria

- Lack of quality or deterioration of medical records;
- Children in primary dentition stage;
- Patients with syndromic alterations or craniofacial malformations;
- Patients with restorations or dental pathology in the molars within the measurements;
- Patients with some treatment with a preformed crown onto temporary molars;
- Patients who have undergone previous orthopedic or orthodontic treatments.

### 2.2. Control Group Population

A—Inclusion Criteria

- Records of children of either sex aged 6 to 12 years;
- Patients with mixed primary and permanent dentition stage;
- Emergence of the first four permanent molars;
- Not having received orthodontic treatment;
- Patients with crowding and diastemas of less than 3 mm and with correct occlusion;
- Class I bilateral molar, 2 to 3 mm overjet and 2 to 4 mm crosslinking.

B—Exclusion Criteria

- Lack of quality or deterioration of medical records;
- Children in primary dentition stage;
- Patients with syndromic alterations or craniofacial malformations;
- Patients with restorations or dental pathology in the molars to be measured;
- Patients with some treatment with a preformed crown onto temporary molars;
- Patients who have undergone previous orthopedic or orthodontic treatments.

### 2.3. Measurement of Study Models

**Figure 1:**
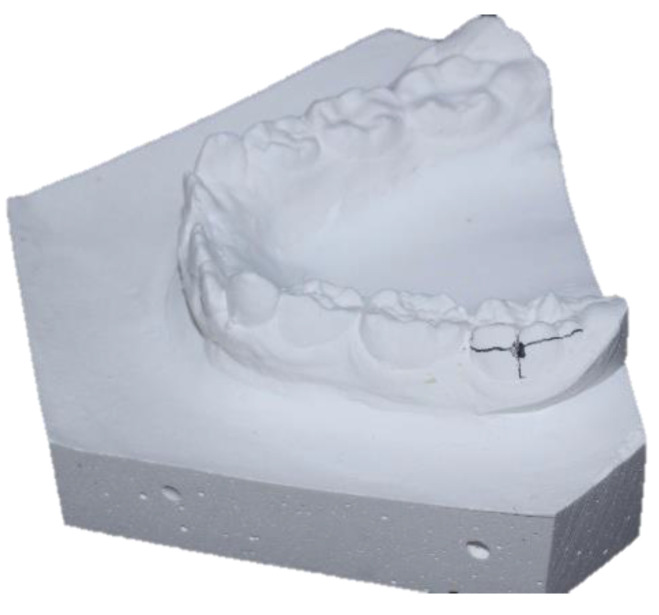
Location of the Axial Face (AF) points. Figure obtained by Dr. Ramon Herrero R.

Once the plaster study models of the maxilla and mandible of each patient were obtained, the Axial Face (AF) points were located in the center of the clinical crown of the first molars and the Wala Ridge points. To locate the AF point at the level of the first molar, it is necessary to measure the width of the molar (mesiodistal diameter) and the height (occlusogingival diameter in the middle of the mesiodistal width), where both measurements intersect is the center of the clinical crown or AF point (Figure 1).

**Figure 2:**
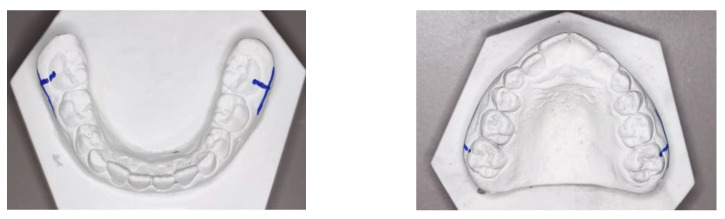
Location of the Wala Ridge points. Figure obtained by Dr. Ramon Herrero R.

Subsequently, the Wala Ridge points were recorded: for this purpose, the projection of the AF points was traced by a tangent to the vestibular side of the molar and perpendicular to the occlusal plane in an occlusal view of the model. This projection will reach the maximum bone contour line, where the Wala Ridge point will be found (Figure 2). 

Once all the points were obtained, and marked with a permanent pen, it was measured with a Mestra^®^ brand digital caliper with a precision of 1 hundredth of a millimeter; the width between the Wala Ridge points of the right and left first permanent molar was measured to obtain the width of the bony bases. Next, it was observed whether there was a maxillomandibular discrepancy (difference in millimeters of maxillary width and mandibular width at the level of the first molar on the line of maximum bone contour) by comparing the upper model with the lower model of each individual. This way, it was checked whether there was a skeletal deficiency of the maxilla in relation to the mandible.

### 2.4. Photo Measurement

The intraoral photographs were always made with the same material and technique:

- The child sitting in the chair is placed straight and with the mouth closed in maximum intercuspation, with the Frankfurt plane parallel to the floor with lateral Ortolan Dental^®^ retractors;
- The camera used was a Reflex Canon^®^ 650D, with a 60 mm Canon^®^ macro lens;
- The brightness was achieved with an ISO of 400, a focal length of F22;
- The focus point was centered on the incisal edge between the upper incisors.

**Figure 3:**
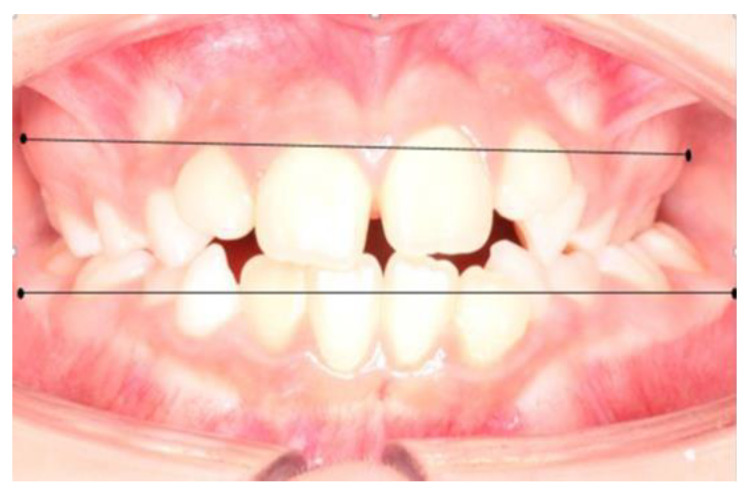
Measurement of maxillary and mandibular width from the location of the Wala Ridge points at the level of the first permanent molar. Figure obtained by Dr. Ramon Herrero R.

The maximum bone contour lines (mucogingival line) superior and inferior were located on the intraoral photograph, marking the most prominent point at the level of the first permanent molars, defining it as the Wala Ridge point (Wr). From the WR point of the first upper right molar to the WR point of the first upper left molar, a line called the maxillary line (Lmx) was drawn, proceeding in the same way in the lower arch, the mandibular line (Lmd). This way, two linear measurements were obtained (Figure 3) which, when subtracted, resulted in the maxillomandibular difference. For the measurement of the upper wala ridge and lower wala ridge distances, the Meazure^®^ measurement program was used.

Once the measurements on the study models and the intraoral photographs were taken, they were both compared.

The ratios between the upper and lower basal distances measured in the photographs with the ratios obtained from the models were calculated at a percentage value. The reason for comparing transversal ratios in percentage values is that the absolute value measurements of the upper and lower basal distances taken in the images are not valid because the photographs are not scaled to the actual size of the mouth. Percentage value ratios are not affected by the scale of the image.

The same operator with an interval of one week repeated all the measurements, in order to calibrate the measurement procedure and obtain intra-operator concordance. If there were any differences in the measurements, the operator did a third measurement and made the mean of the three.

### 2.5. Study Variables

The variables related to sample characteristics were as follows:

- Patient’s sex: Male or female;
- Patient’s age: Ages 6 to 12 years old included;
- Dentition stage: Mixed primary or permanent dentition stage;
- Number of teeth affected: Crossbite of the first permanent molar only, or if additional teeth present crossbite;
- Type of crossbite: Bilateral or unilateral crossbite;
- Crossbite side: Right or left unilateral crossbite.

The variables to be studied were, both in the frontal intraoral photographs and in the study models:

- Maxillary width: Distance from the Wala Ridge point corresponding to the first upper right molar to the Wala Ridge point corresponding to the first upper left molar;
- Mandibular width: Distance from the Wala Ridge point corresponding to the first lower right molar to the Wala Ridge point corresponding to the first lower left molar;
- Maxillomandibular discrepancy: Difference between maxillary width and mandibular width.

### 2.6. Statistical Analysis

To compare the existence of statistically significant differences between the measurement of the maxilla with respect to that of the mandible, assessed by means of intraoral frontal photography or assessed by means of plaster models, the Student’s *t*-test was applied. Likewise, the Pearson or Spearman correlation coefficient was calculated to assess the linear association between the values of both measurements, according to their parametric or non-parametric behavior.

All statistical calculations will be performed using SPSS statistical software (version 23.0 IBM Corp., Armonk, NY, USA). Statistical significance will be considered to exist when the *p*-value is less than the alpha error (5%).

## 3. Results

Regarding the results obtained, starting with the descriptive analysis, we obtained that of the 204 patients; 47.1% were girls and 52.9% were boys with an average age of 9.1 years.

The characteristics of the sample were similar in sex and age of the patient in the control group and in the study group. This was not the case in the dentition stage, since in the control group there was a higher percentage of patients in the first mixed dentition stage, and a higher percentage of patients in the secondary mixed dentition stage in the study group.

Regarding the number of teeth, there was a higher percentage of patients with posterior crossbite, where in addition to the first permanent molar there were more teeth affected. 

Unilateral crossbite was present in 70.5% of patients compared to 25.5% of patients who had bilateral crossbite.

The measurements of the maxillary width in relation to the mandible measured as a percentage value were similar in both the intraoral frontal photograph and in the study models in the control group.

The same was true in the study group: the measurements of the maxillary width in relation to the mandible in percentage value were similar both in the intraoral frontal photograph and in the study models. 

**Figure 4:**
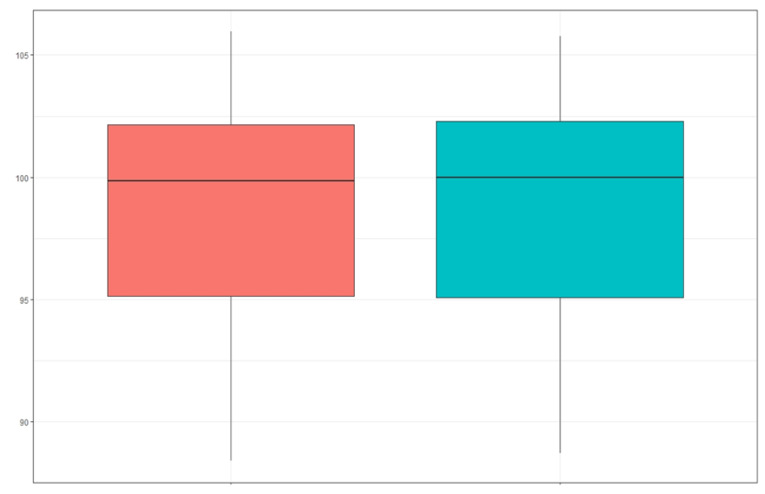
Boxplot of the distribution of the maxillary size data with respect to the mandible in the intraoral frontal photograph and in the study model. The red bloxplot is the results of the photography group, and the green bloxplot is the one for the model group.

**Figure 5:**
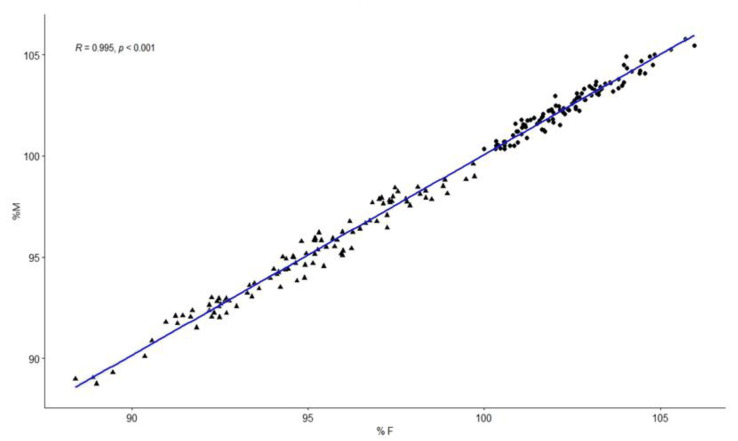
Correlation analysis between the values obtained in the intraoral frontal photograph and those obtained in the study models. The round points are the points of the control group, and the triangle points are the points of the pathology group.

Therefore, there were no statistically significant differences between the measurements taken in the intraoral frontal photographs and the study models; therefore, intraoral frontal photographs can be used as a diagnostic method for posterior crossbite. Correlation analysis revealed a high linear correlation between the two methods (Figure 4 and Figure 5).

**Figure 6:**
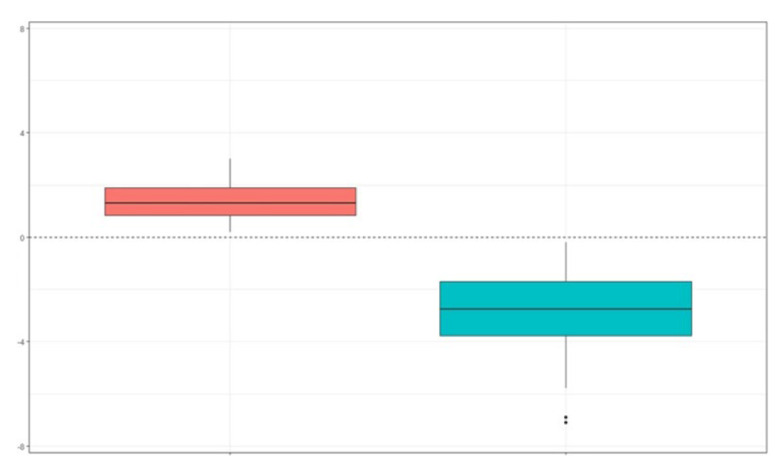
Boxplot of the maxillomandibular difference in the control group and in the study group. The red bloxplot is the control group and the green bloxplot is the pathology group.

There were statistically significant differences in the maxillomandibular difference between the control group and the study group, as in the control group, the maxilla presented a larger size than the mandible in all patients (100%), and in the study group, the maxilla presented a smaller size than the mandible in the totality of the patients. The result obtained for the maxillomandibular difference in the control group was 1.4 ± 0.7 mm, and for the study group it was −2.8 ± 1.4 mm. Therefore, there were statistically significant differences with a *p* < 0.001 (Figure 6).

After analyzing the maxillomandibular difference between sexes, boys and girls, for the variables of the study group, we were able to observe that there were no statistically significant differences in terms of sex. With a *p* > 0.01.

In patients with posterior crossbite, there was less maxillomandibular discrepancy if the crossbite affected only the first permanent molar than if it affected more teeth, significantly with a *p* < 0.05.

Depending on the dentition stage, there were no statistically significant differences in the maxillomandibular difference in patients who were in the mixed primary dentition stage or in the mixed permanent dentition stage. With a *p* > 0.05.

According to the type of crossbite, patients with bilateral crossbite presented greater maxillomandibular discrepancy than patients with unilateral crossbite, therefore, there were statistically significant differences. With a *p* < 0.05. 

Regarding the side of involvement in patients with unilateral crossbite, there were no statistically significant differences between the right and left sides. With a *p* > 0.01.

## 4. Discussion

The importance of a correct diagnosis is key to being able to develop a good method of action. Posterior crossbite generates an imbalance in the stomatognathic apparatus, and although there is literature that reports the self-correction of this pathology, in the transition period between the primary and mixed dentition, this occurs in a very low percentage, 0–10%. Different studies such as those by Petrén, S. [19] and Godoy F. [22] carried out in groups of untreated patients show that self-correction of posterior crossbite does not occur in the transition from mixed dentition to permanent dentition, therefore that a good diagnosis of transverse bone problems is important. This low percentage and the benefits obtained with an early intervention should be sufficient reasons to be able to take action [23].

The exact location of the problem must be known to provide a better diagnosis. According to Haas, [24] differentiating whether the pathology is at the mandibular or maxillary level or both. It may be due to a dentoalveolar alteration or maxillary compression at the apical base. Pure alterations are rare; it is usually the result of a combination of both.

According to Betts et al. [25] in 1995, posterior crossbite is most often associated with a bone problem, and it is not simply a dentoalveolar problem. In agreement with our study, since in 100% of the patients with posterior crossbite there is a negative maxillomandibular discrepancy. 

### 4.1. Diagnosis with Models

Howe et al. [26] proposed a simple rule to predict the arch width by determining an average maxillary intermolar width of 37.4 mm for males and 36.2 mm for females.

McNamara also established a standard in study models of intermolar width of 33–35 mm for children and 36–39 mm for adults [20]. In two review articles by Sawchuck et al. [20] they found that the rule overestimated intermolar distances and predicted maxillary width incorrectly.

In 2010, John L. Hayers [27] proposed a new diagnostic method to determine the center of the alveolar ridge (CAC) and thus measure the maxillary width and mandibular width. In the maxilla, the distance from center to center is measured at the level of the mesial cusps, and in the mandible, at the level of the central fossa. He studied 114 patients aged between 5 and 17 years with a predominance of patients aged between 7 and 9 years. In his study, he concluded that 108 of the patients had maxillary bone pathology. He determined a mean of 40 mm maxillary size and 45 mm mandibular size for males. For females, the mean was 39 mm for the maxilla and 43 mm for the mandible. Due to not taking the same reference points as in our study, the data were different as, in our study, the mean for males was 53.41 mm for maxillary size and 56.1 mm for mandibular size, for females the mean for maxillary size was 51.9 mm and 54.9 mm for mandibular size.

### 4.2. Diagnosis Using the Wala Ridge

L.F. Andrews and W.A. Andrews [15] suggested the use of an anatomical reference as a parameter to centralize the roots of the teeth in the basal bone, which they called the Wala Ridge. In our study, we have used this maximum contour line of both the maxilla and the mandible to analyze the discrepancy between the two jaws. 

Other authors such as Ronay V et al. [28] conducted a study in 2008 to determine if the Wala Ridge was a correct diagnostic method to achieve the ideal arch form for each patient by assessing the gradient between the AF points and the Wala Ridge. The study was conducted by scanning 35 models of the mandibular arches in patients with skeletal Class I, molar and canine. The values they obtained were not similar to those of L. F. Andrews and W. A. Andrews, but they were equally statistically significant. Both confirmed that the Wala Ridge is a reliable method to be used to determine the ideal arch form of patients in advance. 

A similar study was conducted by Ball RL et al. [24], who compared in skeletal class I and skeletal class II patients, the differences between AF points and Wala Ridge points at the canine and first molar level. This was done on plaster models of the mandibular arch, since, as L.F. Andrews and W.A. Andrews said. They concluded that the Wala Ridge points are useful to individually predetermine the arch form for each patient. Therefore, we start from these statements to take the Wala Ridge as a skeletal baseline.

Shu R et al. [29] in 2013, analyzed the bone width of the maxilla and mandible using plaster models, based on the distance from the Wala Ridge point at the level of the first molar. In their analysis they do not study the difference between maxillary and mandibular bone width, but they do indicate the mean bone widths from the line of maximum mandibular and maxillary bone contour (Wala Ridge), these being in patients with Class I bone:

- Mean maxillary bone width at the level of the first molar was 61 ± 1.7 mm;
- Mean mandibular bone width was 57 ± 2.8 mm.

These measurements are similar to those obtained in our study, in which we obtained a maxillary mean of 57.8 ± 1.7 mm and a mandibular mean of 56.4 ± 1.7 mm for the patients in the control group. 

In 2015, Fara Yeste et al. [30] conducted a study on the descriptive analysis of the Wala Ridge in the bone difference between the maxilla and mandible in orthodontics in 74 patients without growth in the permanent dentition (unlike our study which is in the mixed dentition), aged between 29 and 43 years, classifying them into 3 groups:

- G1 patients with Angle Class I bone and teeth, without compression and without malocclusion;
- G2 is formed by individuals with Angle Class I, II and/or III teeth (between 1 and 4 mm), and a dentoalveolar compression between 1 and 4 mm;
- G3 with the presence of skeletal Class I, II and III malocclusion (more than 5 mm) and Angle’s dental malocclusion, and with skeletal compression (more than 7 mm).

There were statistically significant differences between Group 1 and Group 3 in the difference in maxillary and mandibular widths, measured from the Wala Ridge, with a significance of *p* < 0.05. The maxillomandibular discrepancy in Group 1 was 2.2 ± 1.6 mm, similar to that obtained in our study for the control group which was 1.4 ± 0.7 mm.

This discrepancy in Group 3 was −5.1 ± 1.9 mm, the same as the results obtained in our study for the crossbite group, where a negative bone discrepancy is demonstrated, in our case, with a mean of −2.8 ± 1.4 mm, thus indicating that all the patients with posterior crossbite in our sample present a skeletal problem. These data reveal a difference in measurement at the level of the bony base that could be useful in diagnosing whether there is skeletal compression or malocclusion of dental origin in the trans-verse plane.

In 2019, Alessandra Arana et al. [31] conducted a study in which they measured 150 study models in patients without pathology to compare Hayers’ method and Andrews’ method for measuring the transversal component. They found differences in both methods, indicating that Andrews’ Element III presented greater objectivity. In their study, they subtracted 4 mm from the wala–wala distance of both the upper and lower arches. They obtained a maxillary width of 54.6 ± 3.3 mm, and a mandibular width of 52.3 ± 2.6 mm; therefore, their maxillary and mandibular width data are 4 mm less than those of our study, where we obtained a maxillary mean of 57.8 ± 1.7 mm and a mandibular mean of 56.4 ± 1.7 mm for patients in the control group. 

About the treatment of the posterior crossbite several authors have suggested that early treatment of posterior crossbite is necessary to avoid long-term effects on the normal growth of the jaws and teeth [14,15]. The treatment of the posterior crossbite produced favorable changes in the position of the mandible and prevented functional alterations [32]. Otherwise, the lack of treatment can cause alterations in the activity of some muscles of mastication (i.e., the masseter and temporalis muscles) in children and promote craniomandibular disorders in adolescents [9,10].

Patients with bone compression should be treated at the time a maxillomandilar size difference is diagnosed. The treatment of choice should be the one that acts at the bone level. In growing patients, the palatal suture is not yet sealed, so the treatment is simpler and more favorable [20,23,24,25,32].

It is clear for us that the future of the diagnostic methods professional will use are mostly digital treatments, as CBCT, intraoral scanner and facial scanner [33].

It is necessary to be able to diagnose alterations in the transverse plane with accuracy, to know the position of the teeth in relation to their bony bases and the relationship between them. This study allows us to use the Wala Ridge to diagnose whether or not there is a problem in the transverse plane at the bony level. In addition, it provides us with more tools, such as intraoral frontal photography to measure the maxillomandibular discrepancy from the Wala Ridge.

## 5. Conclusions

Our study provides that intraoral frontal photography can be used as a diagnostic method to measure the maxillomandibular difference from the Wala Ridge. The research shows that the maxillary width in the posterior crossbite group is 52.7 mm (SD 3.7) and the mandibular width is 55.5 mm (SD 3.6). The maxillary width in the control group is smaller than the mandibular width. We also find that the maxillomandibular difference in the posterior crossbite group is negative, and in the control group is positive, showing the difference between groups. The data we collected shows that there are no differences in the study group in terms of sex, or dentition stage and in patients with a posterior crossbite, there is less maxillomandibular discrepancy if the crossbite affects only the first permanent molar than if it affects more teeth. We believe that more research should be done in this field in order to help clinicians in the diagnosis of posterior crossbite.

## Data Availability

The data that support the findings of this study are available from the corresponding author, [A.A.], upon reasonable request.
